# Sizing and quantification of micro- and nanoplastics by single particle ICP-MS: a comparison between quadrupole and high-resolution instruments

**DOI:** 10.3389/fchem.2026.1868679

**Published:** 2026-07-22

**Authors:** Pia Leban, Filip Beutels, Géraldine Dumont, Xiaoyu Zhang, Milica Velimirovic, Kristof Tirez, Janja Vidmar

**Affiliations:** 1 Department of Environmental Sciences, Jožef Stefan Institute, Ljubljana, Slovenia; 2 Jožef Stefan International Postgraduate School, Ljubljana, Slovenia; 3 Flemish Institute for Technological Research (VITO), Mol, Belgium; 4 Organic and Biological Analytical Chemistry Group, MolSys Research Unit, University of Liège, Liège, Belgium; 5 Toxicological Center, University of Antwerp (UA), Wilrijk, Belgium

**Keywords:** carbon-based detection, high-resolution ICP-MS, metal-based detection, microplastics, nanoplastics, quadrupole ICP-MS, single particle ICP-MS

## Abstract

Micro- and nanoplastics (MNPs) are widespread environmental pollutants of growing concern due to their persistence and impacts on ecosystems and human health. Their small size, chemical complexity, and low environmental concentrations make accurate detection and quantification challenging. Single-particle (sp) ICP-MS has emerged as a promising technique for sizing and quantifying small MNPs based on their elemental content, yet the performance of different ICP-MS instrument types remains insufficiently evaluated. In this work, the analytical performance of quadrupole (Q) and high-resolution (HR) spICP-MS was systematically compared for sizing and quantifying metal-doped polystyrene (PS) particles at both the micro- (2.5 μm) and nano-scale (198 nm) by monitoring carbon or metal content. For the analysis of NPs via metal-based detection, both instruments enabled the reliable determination of particle size, particle mass, and mass concentration. The HR system showed enhanced metal sensitivity and improved particle size resolution, while the Q system provided better recoveries of particle number concentration. For the analysis of MPs, spICP-HRMS provided superior sensitivity for metal-based detection, whereas spICP-QMS delivered better carbon-based detection performance and lower size detection limits due to its reduced carbon background. Both techniques underestimated particle mass and number concentrations, reflecting the influence of particle losses, especially pronounced in the HR system, and the need for accurate transport efficiency determination. The use of different data processing tools did not have a significant impact on spICP-MS results. These findings emphasize the importance of carefully selecting the type of ICP-MS instrument, detection approach (metal versus carbon), and calibration strategy for reliable quantification and size characterization of MNPs by spICP-MS.

## Graphical Abstract

**Figure:**
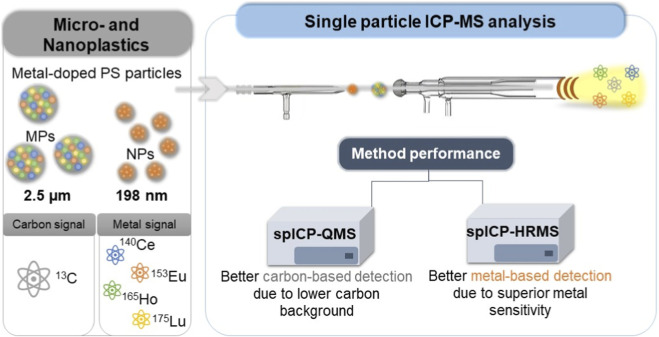

## 1 Introduction

Global plastic production has increased dramatically in recent decades and is currently estimated at approximately 400 million metric tonnes annually (OECD, 2024). Combined with inadequate waste management and the intrinsic resistance of plastics to chemical and thermal degradation, this has led to their widespread accumulation in the environment (da Costa and Opin, 2018). As plastics break down into smaller fragments, they generate microplastics (MPs, particles <5 mm) and nanoplastics (NPs, particles <1 µm) (Frias and Nash, 2019; Gigault et al., 2018), which pose hazards across aquatic, terrestrial, and atmospheric environmental compartments as well as to biota (Frias and Nash, 2019; Gigault et al., 2018). These micro- and nanoplastics (MNPs) can also enter the food chain (Sohail et al., 2023), accumulating in the human body and potentially contributing to various adverse health effects, such as genotoxicity, cytotoxicity, inflammation, and oxidative stress (Winiarska et al., 2024). Their small size and high specific surface area increase the adsorption of environmental contaminants, enhancing their bioavailability and facilitating the crossing of biological barriers and accumulation in tissues and organs (Frias and Nash, 2019; Gigault et al., 2018). Notably, the toxicity of MNPs is expected to increase as particle size decreases, raising further concerns about the impacts of small MPs and NPs (Sharma et al., 2023).

Evaluating the potential risks and hazards of small MPs and NPs requires robust analytical tools capable of their detection, characterization, and quantification. However, analysis of MNPs is hindered by several factors, including their small size, complex and heterogeneous composition, low environmental concentrations, and complex sample matrices that can induce matrix effects (Ivleva, 2021; Silva et al., 2018). Consequently, a range of analytical techniques, primarily based on microscopy, spectroscopy, mass spectrometry, and light scattering, has been used for the determination of MPs particle size, concentration, and composition (González Herrera et al., 2026; Ivleva, 2021; Schwaferts et al., 2019; Zhang et al., 2024). While effective for larger MPs, most of these techniques lack the sensitivity and quantitative capability required for the analysis of small microplastics in the low-µm range (<20 µm) and nanoplastics, underscoring the need for further methodological advancements (Schwaferts et al., 2019).

One promising technique for the detection of small MPs and NPs is inductively coupled plasma-mass spectrometry operated in single particle mode (spICP-MS). This highly sensitive and high-throughput method provides information on particle size, number, and mass concentrations. In spICP-MS, individual particles entering the plasma are vaporized, atomized, and ionized one at a time. This generates discrete, short-duration ion pulses, each corresponding to a single particle, that are measured in a time-resolved acquisition mode. The intensity of each pulse is proportional to the particle’s mass, while the pulse frequency is related to particle number concentration (Laborda et al., 2014). spICP-MS has been widely used for the analysis of inorganic nanoparticles in complex matrices (Resano et al., 2022) and has recently been adapted for the detection of small MPs via monitoring transient ion signals originating from their carbon content (^13^C isotope (Bolea-Fernandez et al., 2020; Caceres et al., 2025; Laborda et al., 2020; Liu et al., 2022; Sakanupongkul et al., 2024; Trujillo et al., 2023) or ^12^C isotope (Gonzalez de Vega et al., 2021; Gonzalez de Vega et al., 2024)). Carbon-based detection can typically resolve MPs down to ∼1–2 µm, while the detection of smaller particles is hindered by elevated carbon background originating from the carbon dioxide in the atmosphere, plasma and/or sample matrix, combined with the relatively low ionization efficiency of carbon (Lab et al., 2022), as well as the difficulty of discriminating plastic-derived carbon from other carbon-containing particulates. An alternative approach involves spICP-MS analysis of laboratory-prepared MNPs with embedded metal tracers, in which metal is chemically entrapped within the polymer particles and serves as a tracer for MNPs detection, enabling indirect detection via metal ion signals (Mitrano et al., 2019). This approach benefits from the high sensitivity and low background signal of the selected metal tracers, typically lanthanides, gold, palladium, indium, etc. (Liu et al., 2024; Yu et al., 2025), and good selectivity, enabling the detection of model NPs (Kutralam-Muniasamy et al., 2024; Liu et al., 2024). However, this approach is not directly applicable to unknown environmental samples unless prior labelling or inherent metal additives are present. Recent studies using metal-doped NPs have demonstrated their suitability for investigating the uptake, fate, transport, and mechanistic behaviour of model NPs in controlled laboratory exposure experiments (Bair et al., 2024; He et al., 2022; Hendriks et al., 2023; Redondo-Hasselerharm et al., 2021; Tran et al., 2024).

For spICP-MS analysis, ICP-MS instruments equipped with different types of mass analysers can be used, including quadrupole (Q), high-resolution (HR), and time-of-flight (TOF) ICP-MS systems. Each configuration utilizes distinct strategies for mass resolution and interference removal, resulting in different analytical performances in spICP-MS analysis. Quadrupole mass analyser exploits chemically-based separation of target ions from interferences, typically using collision or reaction cells (e.g., helium or hydrogen gases), providing moderate mass resolution (∼300), high sensitivity, and low detection limits (Rua-Ibarz et al., 2020). ICP-HRMS employs a magnetic sector with an electrostatic analyser to physically separate target ions from interferences, achieving much higher resolution (300 to >10,000) and sensitivity compared to QMS, ultra-trace detection limits, and improved accuracy in complex matrices (Rua-Ibarz et al., 2020). ICP-TOFMS, on the other hand, employs a time-of-flight mass analyser with the ability to capture the full mass spectrum of each particle in a single measurement, providing moderate to high resolution and rapid data acquisition, making it particularly suitable for complex samples and multi-element analysis of nanoparticles (Harycki and Gundlach-Graham, 2023; Hendriks and Mitrano, 2023; Tian et al., 2025). An ideal ICP-MS instrument for the spICP-MS analysis of MNPs would enable simultaneous multi-element monitoring, without sacrificing sensitivity, and provide high (physical or chemical) resolution. However, no single instrument currently meets all these requirements. While ICP-QMS instruments have so far been the most commonly used for the detection of MNPs in single particle mode (Bolea-Fernandez et al., 2020; Caceres et al., 2025; Gonzalez de Vega et al., 2021; Gonzalez de Vega et al., 2024; Laborda et al., 2020; Liu et al., 2022; Sakanupongkul et al., 2024; Trujillo et al., 2023), they can be limited in carbon-based detection of MPs, primarily due to elevated carbon background, arising mainly from the ubiquitous carbon dioxide, as well as from polyatomic interferences that overlap with the carbon isotopes ^12^C^+^ (^11^B^1^H^+^, ^24^Mg^2+^) and ^13^C^+^ (^12^C^1^H^+^, ^11^B^1^H_2_^+^). These interferences can be mitigated using ICP-HRMS, which can physically resolve carbon ion signals from spectral interferences, potentially enabling more accurate and sensitive detection of small MPs compared to ICP-QMS. However, to date, no studies have reported carbon-based measurements of MNPs using spICP-HRMS. For metal-doped MNPs, the superior sensitivity of ICP-HRMS could further improve detection limits. However, these advantages come with trade-offs, including higher instrument cost and more complex operation compared to quadrupole systems. ICP-TOFMS, with its capability for simultaneous multi-element detection of individual particles, offers clear benefits for metal-doped plastics, enabling the use of multiple tracers to differentiate particle types or monitor several MNP populations in parallel (Baalousha et al., 2024; Hendriks et al., 2023). For carbon detection, ICP-TOFMS provides only moderate resolution and cannot fully resolve carbon from interfering polyatomic species, limiting its sensitivity and suitability for carbon-based spICP-MS analysis of MPs (Harycki and Gundlach-Graham, 2023; Hendriks and Mitrano, 2023; Vonderach et al., 2024).

Despite the growing application of spICP-MS for MNPs analysis, the comparative evaluation of the performance of different instrument configurations remains limited. In this work, we systematically evaluated and compared the performance of quadrupole and high-resolution ICP-MS instruments, the two most widely used and accessible instrument types, for the spICP-MS analysis of metal-doped MNPs. For this purpose, we used the metal-doped polystyrene (PS) microparticles (2.5 µm) labelled with four lanthanides (Ce, Eu, Ho, and Lu) and europium-doped PS nanoparticles (200 nm). Specifically, we compared both instruments in terms of sensitivity, signal-to-background ratio, transport efficiency, particle size resolution, measurement precision, and the accuracy of particle size, particle mass, and particle concentration determinations. The study further assessed the capabilities of metal-based versus carbon-based detection approaches for MP analysis, and compared results obtained using different software for spICP-MS data processing (commercially available versus those provided by ICP-MS vendors).

## 2 Materials and methods

### 2.1 Reagents

The standard of europium (Eu) polystyrene (PS) nanoplastics (NPs) (PS-COOH • Europium Chelate), hereinafter referred to as PS-Eu NPs, was purchased from Bangs Laboratories, Inc. (Fishers, IN, USA). The stock standard of PS-Eu NPs was supplied as a 1% suspension in deionized water, corresponding to 2.322 x 10^12^ particles/mL or 10,000 mg/L of PS NPs, with a nominal diameter of 198 nm. A commercially available monodisperse polystyrene microbeads (PS MPs) standard, with a nominal diameter of 2.5 µm and a particle number concentration of 3.30 × 10^5^ particles/mL, was purchased from Fluidigm, Inc (USA). The PS microbeads were doped with natural-abundance lanthanide isotopes of cerium (^140/142^Ce), europium (^151/153^Eu), holmium (^165^Ho), and lutetium (^175/176^Lu). Manufacturer’s data on lanthanide-doped PS MPs, including calculated metal mass per particle and nominal particle size, were obtained from Merrifield et al. (2018) and are provided in Supplementary Table S1. Prior to spICP-MS analysis, the stock PS MPs and Eu-PS NPs standards were sonicated for 10 min in an ultrasonic bath (Nickel-Electro Ltd. Clifton SW3H, Switzerland) and immediately diluted with ultrapure water in successive steps. A dilution factor of 2.5× was applied for PS MPs, while PS-Eu NPs were diluted by 2.5–5.0 × 10^7^ (depending on the instrument), resulting in a final particle concentration of approximately 10^7^ particles/L. Between each dilution step, suspensions were mixed on a vortex mixer (Vibromix 10, Domel, Slovenia). The final dilutions were prepared in three or four replicates.

Europium ICP standard (1,000 mg/L Eu Certipur® in 2%–3% HNO_3_; Merck, Germany), Rare earth element mix for ICP (50 mg/L in 2% HNO_3_; Fluka Analytical, Sigma-Aldrich, St. Louis, USA), Carbon ICP standard (1,000 mg/L of C in H_2_O; Assurance Grade, SPEX CertiPrep™, NY, USA), and Gold ICP standard (1,000 mg/L Au Certipur® in 7% HCl; Merck, Germany) were used for preparing ionic calibration standards for spICP-QMS analysis. For spICP-HRMS analysis, the Europium ICP Standard (10 mg/L Eu in 2% HNO_3_; Honeywell Fluka, Germany), Lutetium (1,000 mg/L in 2% HNO_3_; Spex Certiprep, NY, USA), Holmium ICP Standard (1,000 mg/L in 2%–3% HNO_3_; Supelco Merck, Germany), Cerium ICP Standard (1,000 mg/L in 2%–3% HNO_3_, Supelco Merck, Germany), Carbon (1,000 mg/L, prepared from dried Potassium hydrogen phthalate for analyses, EMSURE Merck, Germany), and Gold Standard Solution (1,000 mg/L Plasma HIQU 10%–20% HCl, AnalytiChem, Belgium, NV) were used.

To determine the transport efficiency (TE) for NPs, suspensions of gold nanoparticles (Au NPs) were used. For spICP-QMS measurements, AuNPs with a diameter of 51 nm (0.05 g/L Au; NanoComposix, San Diego, CA, USA) were employed, whereas for spICP-HRMS measurements, AuNPs with diameters of 19.9 nm, 30.4 nm, 40.5 nm, 61.1 nm, and 80.3 nm (0.1 g/L AuCl_3_; BBI Solution, Cardiff, UK) were used. To determine the TE for spICP-QMS measurements of MPs, a suspension of BCR-165A, reference material of latex spheres, with a diameter of 2.223 ± 0.013 µm (0.2 g/L latex; Joint Research Centre, Geel, Belgium) was used. For spICP-HRMS analysis, polystyrene spheres with diameters of 1.04, 2.07, and 5.19 µm (101.4 g/L, 101.7 g/L, 104 g/L of PS; Bangs Laboratories, Inc., Fishers, IN, USA) were employed for this purpose.

For microwave-assisted acid digestion of PS-Eu NPs and PS MPs, the Superpure HNO_3_ (67%–69% wt, Carlo Erba Reagents, Italy), H_2_O_2_ (30% wt, Merck KGaA, Darmstadt, Germany), and HCl (30% wt, Merck KGaA, Darmstadt, Germany) were used. Ultrapure water (resistivity of 18.2 MΩcm) was obtained from a Millipore Element apparatus (Millipore, Bedford, MA, USA) and used throughout the work for sample preparations and dilutions.

### 2.2 Single particle ICP-QMS analysis

An Agilent 7900 quadrupole ICP-MS instrument (Agilent Technologies, Santa Clara, CA, USA) was used for spICP-QMS analysis. The sample introduction system consisted of the MicroMist glass concentric nebulizer (Glass Expansion, Australia), a quartz Scott double-pass spray chamber cooled to 2 °C, and a quartz torch injector with an inner diameter of 2.5 mm. Instrumental parameters used for the detection of MPs and NPs are listed in Table 1. Briefly, PS MPs were measured either by monitoring the ^13^C isotope (preferred over ^12^C due to its lower background despite lower abundance, enabling improved signal-to-noise ratio for single-particle detection) or isotopes of lanthanides (^140^Ce, ^153^Eu, ^165^Ho, ^175^Lu), while PS-Eu NPs were measured by monitoring the ^151^Eu isotope. The instrument operated with a dwell time of 100 μs, and data were collected over a total measurement time of 60 s (equivalent to 600,000 data points) for NPs or 115 s (equivalent to 1,150,000 data points) for MPs. Argon gas (purity 99.999%) was used, containing ≤0.05 ppmV of C_n_H_m_.

**TABLE 1:** Instrumental parameters for spICP-QMS and spICP-HRMS analysis of micro- and nanoplastics.

Instrumental parameter	spICP-QMS		spICP-HRMS
Instrument	Agilent 7900		Attom ES
Sample measured	Microplastics	Nanoplastics	Microplastics and nanoplastics
Nebulizer	Concentric (MicroMist, borosilicate glass)		Concentric (borosilicate glass 1 mL/min)
Spray chamber	Scott type, double pass (quartz)		Cyclonic
Torch, id injector	2.5 mm		1.5 mm
RF plasma power	1600 W	1550 W	1300 W
Nebulizer gas flow rate	0.4 L/min	0.7 L/min	32 PSI*
Makeup gas flow rate	0.7 L/min	0.4 L/min	—
Sampling depth	4 mm	8 mm	3.34 mm
Sample flow rate	∼0.030 mL/min	∼0.32 mL/min	∼0.26 mL/min
Integration time	100 μs		50 μs
Measurement time	115 s	60 s	60 s
Isotopes monitored	^13^C, ^140^Ce, ^153^Eu, ^165^Ho, ^175^Lu	^151^Eu	^13^C, ^140^Ce, ^151^Eu, ^165^Ho, ^175^Lu

* Based on the nebulizer gas pressure measurements (in kPa) on the Agilent 7900 instrument, this value would correspond to a flow rate of 0.912 L/min.

Quantification of particle mass for PS MPs based on carbon detection was performed using a calibration curve constructed from a blank (ultrapure water) and four levels of ionic carbon standards (1, 10, 50, and 100 mg/L), prepared in ultrapure water. For lanthanide-based detection, a calibration curve was constructed from a blank (0.1% HNO_3_) and four levels of ionic Ce, Eu, Ho, and Lu standards (0.5, 1, 5, and 10 ng/mL), prepared in 0.1% HNO_3_. Quantification of particle mass for PS-Eu NPs was performed using a calibration curve constructed from a blank (0.1% HNO_3_) and four levels of ionic Eu standards (0.5, 1, 5, and 10 ng/mL), prepared in 0.1% HNO_3_. The TE was determined according to the ʹparticle sizeʹ and ‘particle frequency’ method described by Pace et al. (2011). For MPs, the 2.223 μm latex sphere standard was diluted to a concentration of 2 mg/L, along with ionic carbon standards prepared in ultrapure water. For NPs, the 51 nm Au NPs standard was diluted in ultrapure water to a concentration of 0.125 μg/L, along with ionic Au standards prepared in ICP-True-Rinse solution containing 2% v/v HCl and 0.5% w/v Thiourea (Inorganic Ventures, Inc., Christiansburg, VA, USA). The sample flow rate was accurately determined daily by weighing the amount of ultrapure water collected through the sample introduction tubing over a minimum of 10 min. After each analytical run, the system was rinsed as follows: (1) 10 s probe rinse with 1% HNO_3_; (2) 60 s rinse with 4% HNO_3_; and (3) 60 s rinse with ultrapure water.

### 2.3 Single particle ICP-HRMS analysis

An Attom ES high-resolution ICP-MS (Nu instrument, UK) was used for spICP-HRMS analysis. The sample introduction system consisted of the glass concentric nebulizer with a cyclonic spray chamber and a quartz torch injector with a 1.5 mm inner diameter. Instrumental parameters used for the detection of MPs and NPs are listed in Table 1. Briefly, PS MPs were measured either by monitoring the ^13^C isotope or isotopes of lanthanides (^140^Ce, ^151^Eu, ^165^Ho, ^175^Lu), while PS-Eu NPs were measured by monitoring the ^151^Eu isotope. The instrument operated with a dwell time of 50 μs, and data were collected over a total measurement time of 60 s (equivalent to 1,200,000 data points).

Quantification of particle mass for PS MPs based on carbon detection was performed using a calibration curve constructed from a blank (ultrapure water) and six levels of ionic carbon standards (0.5, 1.0, 2.5, 5.0, 7.5, and 10 mg/L), prepared in ultrapure water. For lanthanide-based detection, a calibration curve was constructed from a blank (1% HNO_3_) and four levels of ionic Ce, Eu, Ho, and Lu standards (0.5, 1, 5, and 10 ng/mL), prepared in 1% HNO_3_. Quantification of particle mass for PS-Eu NPs was performed using a calibration curve constructed from a blank (1% HNO_3_) and four levels of ionic Eu standards (0.5, 1, 5, and 10 ng/mL), prepared in 10% HNO_3_. The TE was determined according to the ʹparticle sizeʹ and ‘particle frequency’ method described by Pace et al. (2011) For MPs, a calibration curve was constructed using 1 µm, 2 µm, and 5 μm PS MP standards diluted in ultrapure water to concentrations of 1.04 mg/L, 2.034 mg/L, and 62.4 mg/L, respectively, together with an ionic carbon calibration curve (for TE determination using Nu Quant software). Alternatively, the 2 μm PS MP standard (2.034 mg/L) was used with an ionic carbon calibration curve for TE calculation in SPCal software. For NPs (TE determined using Nu Quant software), a calibration curve was constructed using Au NP standards of 19.9, 30.4, 40.5, 61.1, and 80.3 nm, diluted to Au concentrations of 0.0649, 0.0649, 0.1299, 0.6494, and 1.2987 μg/L, respectively, together with an ionic gold calibration curve prepared from a 1% HCl blank and four ionic Au standards (0.25, 0.5, 1.0, and 10 ng/mL) in 1% HCl. For TE determination in SPCal software, the 80.3 nm Au NP standard (0.649 μg/L Au) was used in combination with the same ionic gold calibration curve. The sample flow rate was accurately determined daily by weighing the amount of ultrapure water collected through the sample introduction tubing over a minimum of 15 min. After each analytical run, the system was rinsed by running a blank measurement (analysis of ultrapure water).

### 2.4 Data processing

Processing of the results obtained by spICP-QMS was carried out using the Single Nanoparticle Application Module of Agilent (MassHunter 5.3 Workstation software). The software automatically determines the baseline, while the particle detection threshold for discriminating between the particle signals and the background (i.e., instrumental noise and/or dissolved signal) was manually set to the lowest value for which no additional peak would appear on the lower size of the particle size distribution histogram. Results obtained by spICP-HRMS were processed in Nu Quant software. After measurement, the Nu Quant software offers the ability to have a visual control of the detected events. This visual control allows limiting tailing of the event peaks, coinciding events, and saturation events to a minimum by selecting the right smoothing factor of the data. A manual setting of the particle detection threshold offered the ability to choose the lowest discrimination between particle and background signals.

In addition to the vendors’ software built into the ICP-MS instruments, the data obtained from spICP-QMS and spICP-HRMS were also processed using the open-source, Python-based software “SPCal” (Version 1.3.3) (Lockwo et al., 2021). To differentiate single particle events from the ionic background and noise, the thresholding method was set to automatic. Poisson statistics (α = 0.001) were applied by default across all datasets, except for the PS-Eu NPs results obtained from spICP-QMS and the carbon-based results of PS MPs obtained by spICP-HRMS, where Gaussian statistics (σ = 5) were used instead. Additionally, 1, 3, or five points were specified in the advanced thresholding options for the Signal Mean accumulation method. Increasing the threshold to three or five points reduced false-positive detections caused by elevated background noise and fluctuations in the background signal that could otherwise be misidentified as particle events. The one-point thresholding option was applied to Au NPs used for TE determination, whereas three- or five-point thresholds were used for the analysis of MPs and NPs.

For PS-Eu NPs, particle mass obtained by spICP-MS was converted to particle diameter by considering spherical particles, a PS density of 1.06 g/cm^3^, and Eu mass fraction of 0.0149 per particle, which was calculated by using the following equation (Equation 1):

$$Eu\;mass\;fraction=\frac{Mass\;of\;Eu\;per\;particle}{Mass\;of\;PS\;per\;particle}$$ (1)

First, the total mass concentration of Eu in the stock PS-Eu NPs solution was determined by total ICP-MS after microwave-assisted acid digestion. Since spICP-QMS analysis showed that 10.5% ± 0.3% of Eu was present as dissolved ions, the total Eu concentration was corrected to account for this dissolved fraction. The resulting corrected total Eu mass concentration was then divided by the particle number concentration provided by the manufacturer to obtain the mass of Eu per individual particle. Finally, the mass of PS per particle was calculated assuming spherical geometry and the known PS density.

For PS MPs, particle mass obtained via carbon-based detection was converted to particle diameter by considering spherical particles, a carbon mass fraction of 0.923 (corresponding to the mass fraction of carbon in PS), and the density of PS. For lanthanide-based detection, particles were assumed to consist entirely of the lanthanides for the purpose of converting measured lanthanide signals to particle mass. In this case, a mass fraction of 1 was applied for Ce, Eu, Ho, and Lu, using the respective elemental densities of 6.77, 5.25, 8.80, and 9.84 g/cm^3^. Furthermore, particle mass concentration was obtained by multiplying the particle number concentration by the mean particle mass. The method used to calculate TE (either ‘particle size’ or ‘particle frequency’) was selected based on the measurement objective, following the recommendations of Geiss et al. (2022) The ‘particle size’ method was applied when particle size and particle mass were the primary outcome, whereas the ‘particle frequency’ method was used for determining particle number concentration, as it more accurately accounts for particle losses in the sample introduction system. The size limit of detection (LOD) was calculated with SPCal software as follows. First, the signal LOD was determined using a Poisson filter based on Currie’s statistical method. This signal LOD was then converted into a mass LOD either using instrument-specific parameters (TE, ionic response, dwell time, flow rate, and mass fraction) or via a mass response derived from a mean signal of a target element in a standard particle. Finally, the size LOD was derived from the mass LOD assuming spherical particles of known density. The complete calculation procedure is described in detail by Lockwo et al. (2021) The signal-to-background ratio was calculated as the ratio of the mean particle signal intensity to the background intensity. The expanded uncertainties and trueness of the spICP-QMS and spICP-HRMS analyses were determined as described in the Supplementary Material.

### 2.5 Total ICP-MS analysis

The total mass concentration of elements (Eu in PS-Eu NPs and Eu, Ho, Ce, Lu in PS MPs) was determined after microwave-assisted acid digestion of the stock standard suspensions, followed by the ICP-MS analysis. For the PS MPs standard, a 4 mL sample aliquot of a stock suspension was transferred into a Teflon vessel, followed by the addition of 3 mL of HNO_3_, 5 mL of H_2_O_2_, and 2 mL of HCl. The solution was left for 10 min to allow the release of gases, and then subjected to microwave digestion in a CEM MARS six system (CEM Corporation, Matthews, NC, USA). The digestion program consisted of ramping the temperature to 140 °C over 20 min and holding for 2 min, followed by ramping to 200 °C over 15 min and holding at 200 °C for the next 60 min. For the PS-Eu NPs, the stock standard suspension was first diluted with ultrapure water to achieve a PS concentration of 1 mg/L. Subsequently, 10 mL of this solution was transferred to a Teflon vessel, and the same reagents (HNO_3_, H_2_O_2_, and HCl) were added as described above. The solution underwent the same microwave digestion procedure as for PS MPs. After cooling, both digested solutions were transferred to a 50 mL tube and brought to a final volume of 40 mL with ultrapure water. The solutions were further diluted with ultrapure water as needed prior to ICP-MS analysis.

The total element concentrations (Eu, Ho, Ce, Lu) in the PS-Eu NPs and PS MPs samples were determined using an Agilent 7900 or 7700 ICP-MS instrument from Agilent Technologies. The instrument was tuned daily using a multi-element standard solution (1 ng/mL Co, Ce, Y, Li, Mg, Tl in 2% HNO_3_) to optimize the instrument parameters for maximum sensitivity and robustness. The ICP-MS instrumental parameters used for the analysis are summarized in Supplementary Table S2. Quantification was performed based on the external calibration of standards containing Eu, Ce, Ho, and Lu in the concentration range of 0.01–20 ng/mL with online internal standardization (25 ng/mL solution of Ge, Rh, Ir, Bi). Standard calibration solutions were prepared in 2.04% HNO_3_ and 0.6% HCl to match the matrix of the diluted digested samples. Blank samples and control ionic standards (containing 100 ng/mL of Ce, Eu, Ho, Lu) were digested and analysed within each batch alongside the samples to verify the performance of the analytical procedure (Supplementary Table S3).

### 2.6 Py-GC-MS analysis

Polystyrene mass concentration was determined using a microfurnace pyrolyzer (Multi-shot EGA/Py3030-D, Frontier Lab Ltd., Japan) equipped with an auto-shot sampler (AS2020-E, Frontier Lab Ltd., Japan) coupled to a gas chromatograph (7890B, Agilent Technologies, USA) and a single quadruple mass spectrometer (5977B MSD, Agilent Technologies, USA). Pyrolysis was carried out at 590 °C. The resulting pyrolysates were transferred to the GC column (DB-5Q, 30 m × 0.25 mm i.d. x 0.25 μm d.f., Agilent Technologies, USA) using Helium as carrier gas at a constant flow rate of 1 mL/min, with a split ratio of 50:1 and an interface temperature of 300 °C. The GC oven temperature was programmed from 40 °C (held for 2 min) to 320 °C (held for 14 min) at a rate of 20 °C/min. The mass spectrometer was operated in electron ionization (EI) mode at 70 eV, in positive ion mode, with full scan acquisition (m/z 40–550), and a scan rate of 2.9 spectra/s. Chromatograms were analysed using the Agilent MassHunter Qualitative Analysis 10.0 Software (Agilent Technologies, USA).

### 2.7 Nanoparticle tracking analysis

Particle size distribution of PS-Eu NPs was assessed by Nanoparticle Tracking Analysis (NTA) using ZetaView PMX 120 analyser (Particle Metrix GmbH, Germany). Due to the limitations associated with highly concentrated samples, which can interfere with the accuracy of Brownian motion tracking, all water samples were diluted 100,000 times. Before sample measurements, a PS standard (Applied Microspheres, Germany) with a diameter of 100 nm was diluted 250,000 times and used for automatic alignment of the instrument. To prevent cross-contamination between samples, 1–2 mL of ultrapure water (IQ 7000, Merck) was flushed through the system between measurements to ensure complete removal of residual particles.

## 3 Results and discussion

### 3.1 Single particle ICP-MS analysis of nanoplastics

Table 2 summarizes the key performance parameters of the spICP-QMS and spICP-HRMS methods applied for the analysis of PS-Eu NPs, highlighting factors that importantly influence the obtained results. The transport efficiency, determined using both the ‘particle size’ and ‘particle frequency’ methods, was consistently higher for spICP-QMS (7.65% and 7.17%, respectively) than for spICP-HRMS (4.90% and 2.51%, respectively). The difference between TE values obtained by the two approaches is substantially larger for spICP-HRMS, indicating a method-dependent bias in event detection rather than random variability. The particle size approach, which is based on the integrated signal response of detected particle events, is comparatively robust to partial signal attenuation once an event is recorded. In contrast, the particle frequency approach is fundamentally constrained by the completeness of event registration above the discrimination threshold. Under conditions of reduced ion transmission efficiency and higher background variability typical of high-resolution sector field instruments operated at high mass resolution, the probability of missing low-intensity or marginal particle events increases. This leads to systematic undercounting of particle events, and consequently a lower apparent TE calculated based on the ‘particle frequency’ method. Since TE in the spICP-HRMS measurements was determined using AuNPs standards, these observations suggest that the larger discrepancy between the two TE approaches primarily reflects the higher sensitivity of the frequency-based method to counting statistics and event recognition, driven by higher background variability in spICP-HRMS compared to spICP-QMS, rather than actual particle losses during sample introduction. Assuming comparable uncertainty magnitudes for both instruments (Supplementary Table S4), the observed differences between the instruments can be considered statistically significant (p < 0.05, Student’s t-test). This observed discrepancy is most likely related to differences in instrumental configurations and operating conditions between the two instruments (Table 1). Among these factors, the use of make-up gas likely had the greatest impact on the higher TE observed for the spICP-QMS method, as it enhances aerosol transport from the nebulizer to the plasma, reduces particle settling in the spray chamber, minimizes diffusion losses in the sample liner, and improves aerosol focusing in the plasma, thereby increasing the number of particles reaching the ionization region in the plasma (Caceres et al., 2025; Olesik and Gray, 2012). On the contrary, the ionic response was greater in spICP-HRMS for Eu standards, highlighting the superior sensitivity of the HR instrument. As a consequence, the signal-to-background ratio for PS-Eu NPs was substantially higher (8.3-fold) in spICP-HRMS than in spICP-QMS. Accordingly, the particle size detection limit for PS-Eu NPs was lower for spICP-HRMS (81 nm) than for spICP-QMS measurements (100 nm). These results indicate that superior performance for PS–Eu NPs analysis using metal-based detection can be achieved with spICP-HRMS due to the higher sensitivity of the HR instrument, rather than resolution alone.

**TABLE 2:** Performance comparison of spICP-QMS and spICP-HRMS analysis of metal-doped nanoplastics. The reported standard deviation for TE and S/B ratio (spICP-QMS) and for S/B ratio (spICP-HRMS) represents the intra-day repeatability based on three (spICP-QMS) or two (spICP-HRMS) replicate measurements.

Parameter	spICP-QMS	spICP-HRMS
Transport efficiency	Size: 7.65% ± 0.02% Frequency: 7.17% ± 0.03%	Size: 4.90% Frequency: 2.51%
Ionic Eu response	18.97 counts/μg·L^-1^ (R^2^ = 0.9997)	24.32 counts/μg·L^-1^ (R^2^ = 0.9863)
Size limit of detection (nm)	100 nm	81 nm
Signal to background ratio	4,103 ± 22	33,916 ± 1,660

The results obtained from spICP-QMS and spICP-HRMS analysis of PS-Eu NPs, processed using SPCal software (Table 3), show overall good agreement with reference values and consistent precision across all measurands, as demonstrated by intra-day repeatability (Supplementary Table S5) and inter-day reproducibility (Supplementary Table S6). Both instruments provided mean particle diameters (196 ± 1 nm for spICP-QMS and 201 ± 1 nm for spICP-HRMS) that were in close agreement with the reference value provided by the manufacturer (198 nm), although both were higher than the NTA result (184 ± 2 nm). Excellent intra-day repeatability was obtained for both instruments. For particle mass, spICP-HRMS produced a significantly higher value (4,585 ± 21 ag), whereas spICP-QMS yielded a lower value (4,298 ± 50 ag), which was closer to the reference mass (4,306 ag). Particle mass concentrations were underestimated by both techniques relative to the manufacturer’s reference, but agreed well with PS mass concentration determined by Py-GC-MS (100% recovery), with good relative expanded uncertainties. Particle number concentration obtained with spICP-QMS was in close agreement with a reference value, whereas spICP-HRMS yielded a significantly overestimated value (66.3% bias). Because number concentration is directly proportional to event frequency and inversely proportional to TE, the lower apparent TE obtained using the ‘particle frequency’ method results in an overestimation of particle number concentrations, explaining positive bias in the particle number concentrations determined by spICP-HRMS. Inter-day reproducibility of spICP-QMS (Supplementary Table S6) showed increased but still low variability compared to intra-day repeatability, accompanied by modest bias across all measurands.

**TABLE 3:** Characterization of metal-doped polystyrene nanoplastic determined by spICP-QMS and spICP-HRMS. The reported values represent the average ± standard deviation of three (spICP-QMS) or two (spICP-HRMS) replicate measurements performed on the same day.

Technique	Mean particle diameter (nm)	Mean particle mass (ag)	Particle mass concentration (mg/L)	Particle number concentration (particles/mL)
spICP-QMS	196 ± 1	4,298 ± 50	9,197 ± 240	(2.282 ± 0.036) × 10^12^
spICP-HRMS	201 ± 1	4,585 ± 21	9,074 ± 42	(3.862 ± 0.036) × 10^12^
Reference data	198^a^/184 ± 2^b^	4,306^c^	10,000^a^/(9,202 ± 984)^d^	2.322 × 10^12^ ^a^

^a^ As provided by a supplier.

^b^ As determined by NTA.

^c^ Calculated by assuming spherical particles with known particle diameter (198 nm) and polystyrene density (1.06 g/cm3).

^d^ Total PS concentration as determined by Py-GC-MS.

The results presented in Table 3 were also compared with those obtained using the vendors’ software (Supplementary Table S7). The results derived from the vendors’ software were within the expected range of experimental uncertainty for particle size, particle mass, and particle mass concentration. For particle number concentration, the values obtained using the vendors’ software were significantly higher for spICP-QMS than those obtained with SPCal, primarily due to the differences in the calculated TE between the two programs. When applying the ‘particle frequency’ method, the TE calculated by the software of the spICP-QMS instrument was lower (6.85 %± 0.05%; Supplementary Table S8) than that obtained using SPCal software (7.17% ± 0.03%; Table 2) due to the different approaches used for the particle detection threshold determination. For spICP-HRMS, the particle number concentrations obtained by different data processing software were not statistically different, as the TE calculated using the ‘particle frequency’ method was comparable between the two software. These findings indicate that data processing tools integrated within ICP-MS software yield results consistent with those obtained using SPCal, and can therefore be considered reliable for the processing of spICP-MS data for metal-doped NPs, provided that harmonization of the threshold criteria is ensured, making both approaches comparable to SPCal.

**FIGURE 1:**
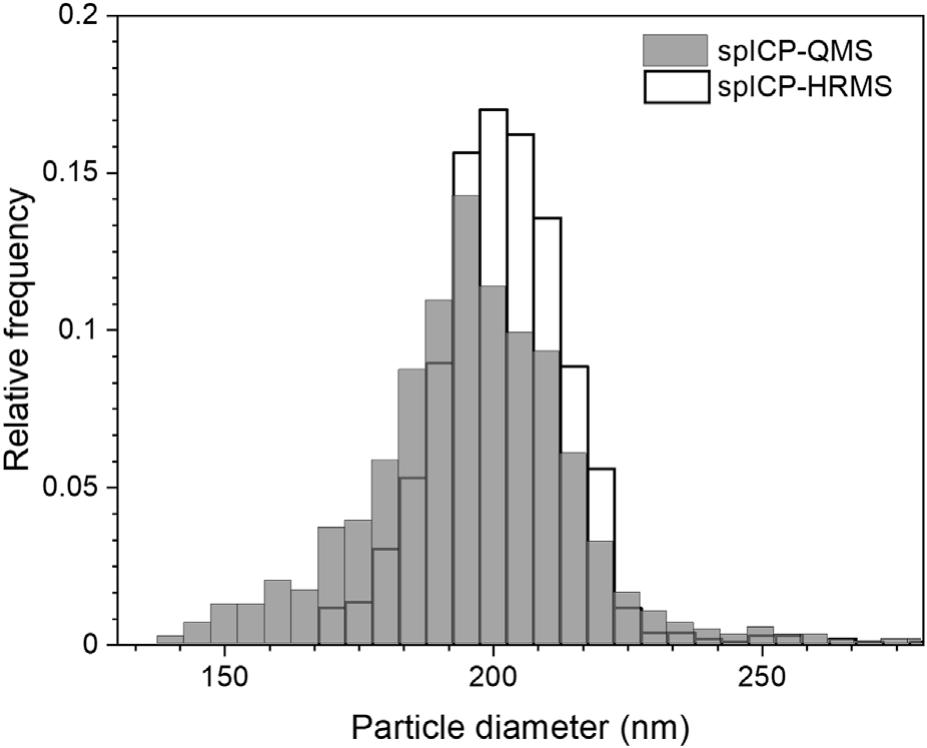
Number-based particle size distribution of Eu-doped polystyrene NPs obtained by using spICP-QMS and spICP-HRMS (bin size 5 nm). The relative frequency was calculated as the ratio between the number of particles counted in a specific bin size and the total number of particles in the sample.

Furthermore, the number-based particle size distributions of PS-Eu NPs obtained by spICP-QMS and spICP-HRMS were compared (Figure 1). Both techniques show a normal size distribution centered around particle diameters ranging from 190 to 200 nm, with spICP-HRMS producing a narrower and more symmetric distribution. The broadening of the size distribution in spICP-QMS can be attributed to greater diffusion of the ion cloud, which is typically more pronounced when using a torch equipped with a larger injector diameter. In this study, injector diameters of 2.5 mm (spICP-QMS) and 1.5 mm (spICP-HRMS) were employed. Consequently, in spICP-QMS, desolvation of droplets with different initial sizes may occur at varying times and spatial locations within the plasma, leading to increased variability in signal intensity and pulse duration for particles of similar sizes and ultimately resulting in a broader size distribution (Laborda et al., 2023; Aghaei and Bogaerts, 2016). In addition, differences in ion extraction, transmission, and detection characteristics between the two mass analyzers may also contribute, to some extent, to the observed differences in distribution width.

Overall, the results show that both spICP-MS instruments provide comparable performance for the quantification and size characterization of metal-doped NPs, showing robust, reliable, and reproducible measurements. Although moderate systematic bias was observed, most notably the overestimation of particle number concentration by spICP-HRMS, these deviations should be considered when interpreting quantitative results. Nevertheless, the associated uncertainties for all measurands remain well within ranges generally considered acceptable for nanoparticle analysis by spICP-MS (Geiss et al., 2022; Montoro Bustos et al., 2018; Yamashita et al., 2023). Although the ICP-HRMS system exhibited higher sensitivity for ionic Eu, along with an improved S/B ratio and lower size LOD for PS–Eu NPs compared to ICP-QMS (Table 2), this did not result in a significant improvement in the overall performance for the analysis of 200 nm PS–Eu NPs. Nevertheless, these advantages of HR instruments may be particularly beneficial for smaller metal-doped nanoparticles, especially those approaching 100 nm in diameter.

### 3.2 Single particle ICP-MS analysis of microplastics

Table 4 compares the performance parameters of the spICP-QMS and spICP-HRMS methods applied for the analysis of metal-doped PS MPs via the detection of carbon and lanthanide isotopes. The TE values reveal substantial differences between the two ICP-MS systems. For spICP-QMS, TE determined using both the ‘particle size’ (10.70%) and ‘particle frequency’ (10.92%) method was higher than that obtained for spICP-HRMS, where TE dropped to 6.50% (according to the ‘particle size’ method) and to 0.819% (according to the ‘particle frequency’ method). The observed differences in TE between the two instruments are likely driven by differences in the sample introduction systems rather than by the mass analyzer type, with the distinct spray chamber designs (double-pass in the spICP-QMS versus cyclonic in the spICP-HRMS) being a particularly relevant factor. Moreover, the instrumental parameters in spICP-QMS were specifically optimized for the detection of larger particles in the micrometre size range. One key factor is the lower sample uptake flow rate used in spICP-QMS (∼0.030 mL/min), which strongly affects particle transport efficiency, with lower uptake rates promoting enhanced solvent evaporation in the spray chamber, thereby improving analyte transport (Lomax-Vogt et al., 2025; Olesik et al., 2021). Consequently, this reduced uptake flow likely may contribute to the higher TE observed for spICP-QMS. In addition, the use of carrier gas flow rate (0.4 L/min) in combination with a make-up gas (0.7 L/min) can reduce aerosol transport losses and enhance particle transfer to the plasma, which may further explain the higher TE observed with spICP-QMS (Caceres et al., 2025; Gonzalez de Vega et al., 2021; Laborda et al., 2020; Liu et al., 2022). In contrast, the spICP-HRMS operating conditions applied for microparticle analysis were not specifically optimized for particles in the micrometre size range, as the same conditions used for Eu-PS nanoparticle measurements were applied. Therefore, a direct comparison between the two systems should be interpreted with caution. The large discrepancy between the size- and frequency-based TE estimates for spICP-HRMS indicates significant losses of the number of PS MPs, either occurring in the stock suspension as a result of particle aggregation or arising from settling during sample dilution or transport to plasma (Geiss et al., 2022; Pace et al., 2011). A systematic underestimation of the ‘particle frequency’-based TE in comparison to the ‘particle size’-based approach has already been reported in spICP-MS analysis (Caceres et al., 2025; Geiss et al., 2022; Loula et al., 2019). Nevertheless, ‘particle frequency’-based TE was deliberately applied for the calculation of particle number concentrations, as it provides a conservative estimate that implicitly accounts for particle losses occurring during sample handling and transport into ICP-MS.

**TABLE 4:** Performance comparison of spICP-QMS and spICP-HRMS analysis of metal-doped microplastic. The reported standard deviation for TE and S/B ratio (spICP-QMS) and for S/B ratio (spICP-HRMS) represent the intra-day repeatability based on four (spICP-QMS) or three (spICP-HRMS) replicate measurements.

Parameter	spICP-QMS	spICP-HRMS
Transport efficiency	Size: 10.70% ± 0.03%	Size: 6.50%
	Frequency: 10.92% ± 0.51%	Frequency: 0.819%
Ionic sensitivity	C: 0.0007 counts/μg·L^-1^ (R^2^ = 0.9988)	C: 0.0023 counts/μg·L^-1^ (R^2^ = 0.9985)
	Ce: 24.44 counts/μg·L^-1^ (R^2^ = 0.9988)	Ce: 37.07 counts/μg·L^-1^ (R^2^ = 0.9892)
	Eu: 17.02 counts/μg·L^-1^ (R^2^ = 0.9982)	Eu: 23.80 counts/μg·L^-1^ (R^2^ = 0.9946)
	Ho: 34.65 counts/μg·L^-1^ (R^2^ = 0.9990)	Ho: 55.80 counts/μg·L^-1^ (R^2^ = 0.9926)
	Lu: 36.41 counts/μg·L^-1^ (R^2^ = 0.9986)	Lu: 58.89 counts/μg·L^-1^ (R^2^ = 0.9927)
Size limit of detection (nm)	656 nm (C), 10 nm (Ce), 12 nm (Eu), 8 nm (Ho), 8 nm (Lu)	916 nm (C), 10 nm (Ce), 13 nm (Eu), 8 nm (Ho), 8 nm (Lu)
Signal to background ratio	C: 198 ± 10	C: 81 ± 3
	Ce: 151,524 ± 9,629	Ce: 611,325 ± 70,450
	Eu: 257,496 ± 71,339	Eu: 757,674 ± 152,779
	Ho: 169,418 ± 55,131	Ho: 795,197 ± 148,680
	Lu: 179,536 ± 40,976	Lu: 333,229 ± 23,692

The ionic response for all lanthanide and carbon isotopes was greater in spICP-HRMS, again reflecting the intrinsically higher ion transmission of the HR analyser. Particle size LOD values for MPs when monitoring metal tracers were nearly identical between the two techniques (8–13 nm), although S/B ratios were substantially higher in spICP-HRMS (up to ∼4.7-fold for Ho and ∼3-fold for Eu) compared to spICP-QMS. The higher sensitivity of ionic carbon in spICP-HRMS (0.0023 counts/μg·L^-1^) compared to spICP-QMS (0.0007 counts/μg·L^-1^) confirms improved ionization and detection efficiency for low-mass analytes, albeit at the expense of increased carbon background arising mainly from the ubiquitous carbon dioxide, as seen in the 1.3-fold lower S/B ratio compared to spICP-QMS. Much lower carbon background in spICP-QMS is a result of a lower sample uptake and nebulizer flow rates, which significantly improves the S/B ratio for carbon despite the instrument’s lower sensitivity (Supplementary Figure S1). This is also reflected in substantially smaller size LOD (656 nm) for PS MPs achieved by spICP-QMS compared to spICP-HRMS (916 nm). These findings indicate that the presence of carbon dioxide in the atmosphere, plasma, and sample matrix contributes far more to the elevated carbon background than polyatomic interferences overlapping the ^13^C^+^ signal, such as ^12^C^1^H^+^ and ^11^B^1^H_2_^+^, which can be effectively resolved by spICP-HRMS.

The characterization data of a lanthanide-doped PS MPs standard obtained using spICP-QMS and spICP-HRMS and processed with SPCal software are presented in Table 5, together with the corresponding bias, intra-day repeatability (Supplementary Table S9), and inter-day reproducibility (Supplementary Table S10). For the analysis of PS MPs based on lanthanide detection, mean particle diameters were generally in good agreement with the reference data. spICP-HRMS yielded significantly smaller mean particle diameters via Ce detection (101.6 ± 0.5 nm) relative to spICP-QMS (109.5 ± 0.4 nm). Regardless of the instrument, Eu exhibited substantial positive bias (21%), while Ho and Lu consistently underestimated particle diameter (−4.7% to −8.5%). Similar trends were observed for particle mass, although the accuracy for Ce differed to a greater extent between instruments (1.3% bias for spICP-QMS versus −18.7% for spICP-HRMS). Detection based on the Eu signal resulted in significantly higher recovered particle mass compared to the reference data (76.0%–79.8% bias), whereas monitoring the Ho and Lu signals consistently underestimated particle mass (−12.6% to −23.1%). It should be noted that the reference values for particle diameter and mass were derived from literature data, as the manufacturer of the MPs standard provided only the particle number concentration and nominal PS particle size. When the particle mass concentration calculated from the manufacturer-provided data (Supplementary Table S1) was compared with total element concentrations obtained by ICP-MS (Table 5), the largest discrepancy was observed for Eu, for which the measured concentration was more than 50% higher than the theoretical value. If the experimentally determined Eu mass (4545 ag) is considered as the reference, the bias in particle mass decreases to 9.7% (spICP-QMS) and 12.1% (spICP-HRMS). Furthermore, particle mass concentrations measured by spICP-QMS were consistently higher across all lanthanides, although both techniques exhibited strong negative bias for all metal tracers. In contrast, spICP-HRMS consistently yielded higher particle number concentrations, while still showing systematic underestimation. While biases for particle mass concentration and particle number concentration were comparable for spICP-QMS, a substantial discrepancy between particle mass concentration (−88.7% to −91.4%) and particle number concentration (−18.4% to −28.0%) biases was observed for spICP-HRMS. This difference arises from the role of TE in the calculations of these two measurands. Particle number concentration is directly proportional to TE, such that underestimation of TE leads to proportional overestimation of particle number concentration, and vice versa. In contrast, for particle mass concentration, the effect of TE is largely compensated because it is consistently applied in both signal-to-mass conversion and particle counting, rendering the result relatively insensitive to the specific TE value used. In this study, TE determined using the “particle frequency” method was applied for particle number concentration calculations, following recommendations from Geiss et al. (2022), to better account for particle losses during sample introduction and improve accuracy. For this purpose, MPs of a similar size range (∼2 µm) to the analyte PS MPs were used, as appropriate calibrant selection is critical for accurate TE determination (Suárez Priede et al., 2025). The low TE value obtained (0.819%) led to overestimation of particle number concentration relative to particle mass concentration. When TE values derived from the “particle size” method (6.50%) were applied instead, particle number concentration recoveries obtained by spICP-HRMS decreased to 7%–8%, consistent with those for particle mass concentration.

**TABLE 5:** Characterization of metal-doped polystyrene microplastic determined by spICP-QMS and spICP-HRMS. The reported values represent the average ± standard deviation of four (spICP-QMS) or three (spICP-HRMS) replicate measurements.

Element	Technique	Mean particle diameter (nm)	Mean particle mass (ag)	Particle mass concentration (μg/L)	Particle number concentration (particles/mL)
Ce	spICP-QMS	109.5 ± 0.4	4,687 ± 40	0.659 ± 0.057	137,914 ± 13,081
	spICP-HRMS	101.6 ± 0.5	3,760 ± 52	0.118 ± 0.005	249,847 ± 9,311
	Reference data	109.7^a^	4,626^a^	1.375 ± 0.064^c^	330,000^a^
Eu	spICP-QMS	121.9 ± 0.1	4,986 ± 12	0.743 ± 0.052	146,076 ± 10,443
	spICP-HRMS	122.4 ± 0.3	5,095 ± 38	0.153 ± 0.005	237,724 ± 9,340
	Reference data	101.1^a^	2,833^a^	1.547 ± 0.045^c^	330,000^a^
Ho	spICP-QMS	70.7 ± 0.1	1,631 ± 9	0.235 ± 0.011	141,213 ± 6,849
	spICP-HRMS	73.2 ± 0.4	1,820 ± 29	0.062 ± 0.002	269,350 ± 3,944
	Reference data	76.8^a^	2,082^a^	0.549 ± 0.022^c^	330,000^a^
Lu	spICP-QMS	75.1 ± 0.2	2,190 ± 17	0.319 ± 0.021	142,625 ± 9,691
	spICP-HRMS	75.1 ± 0.2	2,208 ± 21	0.071 ± 0.005	254,064 ± 14,965
	Reference data	82.1^a^	2,848^a^	0.794 ± 0.007^c^	330,000^a^
C	spICP-QMS	2,890 ± 22	(1.44 ± 0.03) × 10^7^	2,185 ± 180	149,849 ± 9,255
	spICP-HRMS	2,940 ± 42	(1.45 ± 0.06) × 10^7^	468 ± 49	255,118 ± 18,275
	Reference data	2,500^a^	8.67 × 10^6^ ^b^	2,860^d^/(1,837 ± 82)^e^	330,000^a^

^a^ As provided by a supplier/literature data.

^b^ Calculated by assuming spherical particles with known particle diameter (2500 nm) and polystyrene density (1.06 g/cm3).

^c^ Total element concentration as determined by conventional ICP-MS analysis of the acid-digested stock solution.

^d^ Calculated by multiplying the particles number concentration as provided by a supplier with the mean particle mass.

^e^ Total PS concentration as determined by Py-GC-MS.

The relative expanded uncertainty of PS MP analysis by spICP-QMS and spICP-HRMS, assessed for intra-day (Supplementary Table S9) and inter-day (Supplementary Table S10) measurements, was strongly dependent on the selected metal tracer, remaining low for particle size, modest for particle mass, and moderate for both particle mass concentration and particle number concentration. Inter-day measurements indicated that instrumental drift further increased the underestimation of particle size for Ho and Lu, with a similar trend observed for particle mass. For particle mass concentration, combining data across multiple days increased overall uncertainty and improved precision, while particle number concentration also exhibited enhanced precision across multiple days compared with single-day measurements.

For carbon-based detection of PS MPs, a similar trend was observed. While similar mean particle diameter and particle mass were obtained by both spICP-HRMS and spICP-QMS, they were systematically overestimated with biases of 15.6%–17.6% for particle diameter and 64.6%–67.6% for particle mass. SpICP-HRMS resulted in substantially lower particle mass concentrations, while reporting higher particle number concentrations compared to spICP-QMS. Consistent with observations for lanthanide-based detection, particle losses within the spICP-HRMS system were more pronounced than for spICP-QMS, leading to substantial underestimation of particle mass concentrations. This underestimation was more pronounced than for particle number concentration, as the low TE determined using the “particle frequency” method partially compensated for particle losses in the calculation of particle number concentration. Precision for all measurands was generally good, with relative expanded uncertainties remaining low for particle size, modest for particle mass, and moderate for both particle mass concentrationand number concentration. These results indicate that particle size can generally be determined with good reproducibility and only minor systematic deviation, whereas particle mass, mass concentration, and number concentration are subject to considerably greater uncertainty and bias. This reflects inherent limitations in the detection of MPs by spICP-MS, including variations in particle transport behaviour and incomplete particle detection, both of which contribute to systematic bias.

The results presented in Table 5, processed with SPCal software, were further compared with those obtained by the vendors’ software (Supplementary Table S11). For spICP-QMS, particle size, particle mass concentrations, and particle number concentrations were comparable between the two data processing tools across all measured isotopes, with variations remaining within the expected range of measurement uncertainty. For particle mass, the discrepancies exceeded the expected range of experimental uncertainty for all elements but remained within the 4.5% of the average value. These variations may be attributed to differences in the calculated TE between the two software, with the TE obtained using the ‘particle size’ method being 0.25% lower in the vendor’s software (Supplementary Table S8). Similarly, for spICP-HRMS, particle mass concentrations and particle number concentrations obtained using vendor software were not statistically different from those calculated with SPCal, whereas particle size and mass were consistently higher across all measured isotopes when vendor software was applied. This difference is attributable to the higher TE determined using the “particle size” method in the vendor software (8.95%; Supplementary Table S8) compared to SPCal (6.50%), which directly influences the calculated particle mass and, consequently, the cube root–derived particle diameter. The discrepancy in TE values between the two data processing approaches likely arises from differences in calibration strategy, as the Nu Quant software employed a multi-point calibration using 1 µm, 2 µm, and 5 μm PS MP standards, whereas SPCal relied on a single 2 μm PS MP standard for TE determination.

**FIGURE 2:**
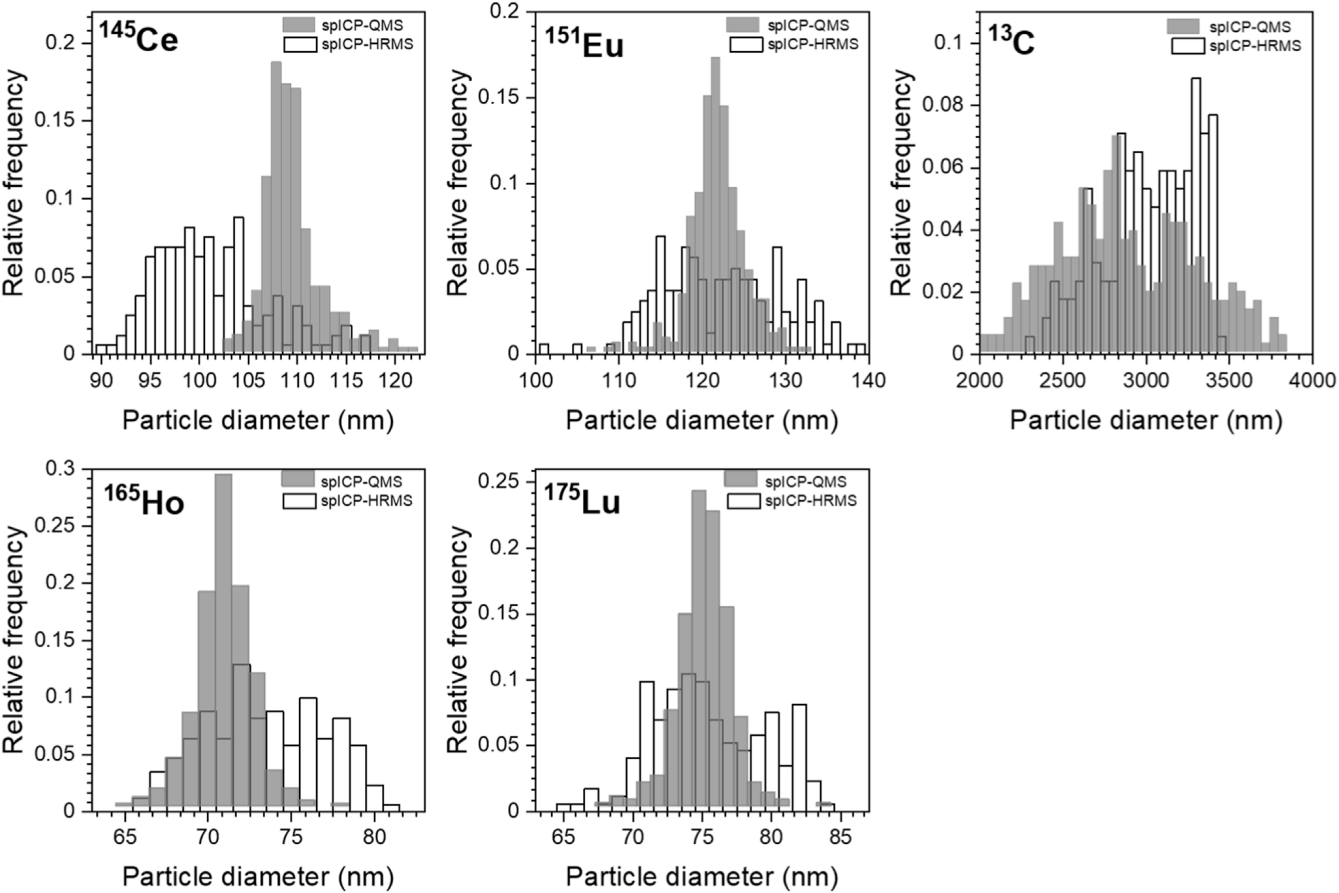
Number-based particle size distributions of lanthanide-doped polystyrene microplastics determined by spICP-QMS and spICP-HRMS (bin size of 1 nm for Ce, Eu, Ho, Lu, and 50 nm for C). The relative frequency was calculated as the ratio between the number of particles counted in a specific bin size and the total number of particles in the sample.

Furthermore, the number-based particle size distributions of PS MPs obtained by spICP-QMS and spICP-HRMS are shown in Figure 2. When monitoring lanthanide elements (Ce, Eu, Ho, and Lu), spICP-QMS showed narrower particle size distributions, while spICP-HRMS produced broader distributions. This effect may be related to the fact that spICP-QMS measurement parameters were specifically optimized for the detection of larger MP particles, resulting in reduced ion cloud diffusion in the plasma, producing a more focused ion stream, which in turn leads to narrower particle size distributions (Caceres et al., 2025; Laborda et al., 2023). In the case of carbon-based detection, both methods showed broader particle size distributions with a wider particle size range. This broadening, previously reported for PS MPs by Laborda et al. (2020), can be attributed to the diffusion of the ion cloud, a phenomenon that is particularly pronounced for analytes with low mass, such as carbon. The addition of make-up gas at the expense of lowered nebulizer gas also enhanced the broadening of the size distribution in spICP-QMS, as reported by Caceres et al. (2025), with a shift toward smaller particle sizes indicating a higher abundance of lower particle intensities due to lower size LOD in spICP-QMS (656 nm) compared to spICP-HRMS analysis (916 nm). The asymmetrical size distribution observed for carbon-based detection of MPs by spICP-HRMS may indicate losses of larger particles in the sample introduction system, consistent with the lower particle mass concentration recoveries obtained with spICP-HRMS.

In summary, both instruments provided particle diameter and mass values close to the reference values when monitoring Ce, Ho, and Lu, but overestimated these parameters for Eu and C isotopes. The findings highlight the superior sensitivity of the HR instrument for metal-doped MPs when monitoring lanthanide elements. Conversely, under the instrumental parameters used in this study, spICP-QMS remains more suitable for carbon-based MPs detection due to improved size detection limits and better S/B ratios, as previously reported by Suárez Priede et al. (2025) Particle number and mass concentrations were consistently underestimated by both techniques, regardless of the monitored isotope, reflecting systematic particle losses, likely occurring during sample introduction to ICP-MS or as a result of particle aggregation, either in the stock solution or during sample dilutions. Overall, spICP-HRMS demonstrated superior particle number detection across all isotopes, while carbon-based spICP-QMS analysis yielded PS particle mass concentrations closer to the expected values, suggesting that carbon-based detection may be more reliable for larger MP particles under the selected instrumental conditions.

## 4 Conclusion

Both techniques, spICP-QMS and spICP-HRMS, proved suitable for the reliable quantification and size characterization of metal-doped NPs. The high-resolution ICP-MS demonstrated significantly enhanced sensitivity, improved signal-to-background ratios and size resolution, offering superior accuracy for particle mass and size. In contrast, spICP-QMS yielded slightly better estimates of particle number concentration, primarily due to its higher transport efficiency. Differences between particle size- and particle frequency-based TE were more pronounced for spICP-HRMS and are attributed to the higher sensitivity of the frequency-based approach to counting statistics and event recognition under increased background variability and lower ion transmission efficiency, rather than to actual particle losses during sample introduction. Given the minimal spectral interferences associated with the monitored lanthanide isotopes, the instrument’s performance was primarily influenced by its sensitivity. However, for the relatively large PS-Eu NPs investigated in this study (median diameter of 200 nm), the difference in size detection limits between instruments (81 nm for spICP-HRMS vs. 100 nm for spICP-QMS) is not critical, indicating that extreme sensitivity-driven tuning is unnecessary when monitoring lanthanide isotopes in particles of this size range. Nevertheless, the advantages of HR instruments may be beneficial for smaller metal-doped nanoparticles, especially those approaching 100 nm in diameter.

For the analysis of metal-doped PS MPs, spICP-HRMS showed higher sensitivity, particularly for lanthanide detection. In contrast, spICP-QMS provided superior carbon-based detection due to its lower carbon background and improved signal-to-background ratio, which, despite its overall lower sensitivity, resulted in improved size LOD for carbon. Both techniques underestimated particle mass and number concentrations, suggesting systematic particle losses occurring during sample introduction or as a result of particle settling in the sample itself. Overall, spICP-HRMS achieved more reliable particle number quantification, while carbon-based spICP-QMS produced mass concentration values that were closer to the reference values. Reliable number quantification of MNPs is significantly influenced by the accurate determination of transport efficiency. The spICP-QMS technique showed higher transport efficiency for both nanoparticles and microparticles compared to the spICP-HRMS. This difference was even more pronounced when transport efficiency was calculated using the ‘particle frequency’ method and for MPs. It is important to note that the observed differences are largely governed by differences in the sample introduction system and instrumental operating parameters, such as sample flow rate and make-up gas addition, rather than by the mass analyzer type. The findings of this study underscore the importance of carefully selecting the appropriate ICP-MS instrument, detection approach (metal versus carbon), and calibration strategy for reliable quantification and size characterization of MNPs by spICP-MS, ensuring that they are tailored to the particle size, material composition, and specific analytical objectives.

While spICP-MS offers strong potential for sizing and quantifying MNPs through both carbon-based and intrinsic metal-based particle characterization, unambiguous identification of plastic particles in complex environmental matrices remains challenging. For carbon detection, careful data interpretation is required to distinguish plastic-derived signals from other carbon-containing materials. Metal-based detection, in turn, is not directly applicable to unknown environmental samples unless particles contain intrinsic metal additives or have been deliberately labelled. Consequently, complementary analytical techniques are still necessary to achieve definitive identification of plastic particles.

## Data Availability

The original contributions presented in the study are included in the article/Supplementary Material, further inquiries can be directed to the corresponding author.
