# Supplementary material for: Sizing and quantification of micro- and nanoplastics by single particle ICP-MS: a comparison between quadrupole and high-resolution instruments
Source: Front Chem. 2026 Jul 22;14:1868679. doi: 10.3389/fchem.2026.1868679 (PMC13437643; doi:10.3389/fchem.2026.1868679)
Supplement: Supplementary file 1 [file DataSheet1.docx]

Supplementary Material

**Sizing and Quantification of Micro- and Nanoplastics by Single Particle ICP-MS: A Comparison Between Quadrupole and High-Resolution Instruments**

**Pia Leban^1,2^, Filip Beutels^3^, Géraldine Dumont^3,4^, Xiaoyu Zhang^3,5^, Milica Velimirovic^3^, Kristof Tirez^3^, Janja Vidmar^1,2^***

^1^Department of Environmental Sciences, Jožef Stefan Institute, Jamova cesta 39, 1000 Ljubljana, Slovenia

^2^Jožef Stefan International Postgraduate School, Jamova cesta 39, 1000 Ljubljana, Slovenia

^3^Flemish Institute for Technological Research (VITO), Boeretang 200, 2400 Mol, Belgium

^4^Organic and Biological Analytical Chemistry Group, MolSys Research Unit, University of Liège, 4000 Liège, Belgium

^5^Toxicological Center, University of Antwerp (UA), Universiteitsplein 1, 2610 Wilrijk, Belgium

*** Correspondence:**
Janja Vidmar
[janja.vidmar@ijs.si](mailto:janja.vidmar@ijs.si)

Table S1: Data provided for lanthanide-doped PS MPs (Fluidigm), showing the estimated number of metal atoms per microbead, calculated metal mass per particle, nominal particle size (all obtained from Merrifield et al.^1^), and mass concentration of lanthanides, calculated by multiplying the particle number concentration (330,000 particles/mL) with the provided metal mass per particle.

| **Element** | **Atoms per bead (± 15%)** | **Mass (ag)** | **Size (nm)** | **Mass concentration (µg/L)** |
| --- | --- | --- | --- | --- |
| Ce 140 | 19.9E6 | 4,626 | 109.7 | 1.527 |
| Eu 151 | 11.3E6 | 2,833 | 101.1 | 0.935 |
| Eu 153 | 12.0E6 | 3,049 | 103.6 | 1.006 |
| Ho 165 | 7.6E6 | 2,082 | 76.8 | 0.687 |
| Lu 175 | 9.8E6 | 2,848 | 82.1 | 0.940 |

1. R. C. Merrifield, C. Stephan and J. R. Lead, *Environ. Sci. Technol.*, 2018, **52**, 2271–2277.

**Calculation of expended uncertainties and trueness**

The expanded uncertainties of the spICP-QMS and spICP-HRMS analysis were estimated from replicate measurements performed within a single day to assess intra-day repeatability, while inter-day reproducibility for spICP‑QMS was evaluated using replicate measurements collected over multiple days.^1^ Standard uncertainties ($u_{A}$, Equation S1) were first calculated for each measurand (particle size, particle mass, particle mass concentration, and particle number concentration), with expanded uncertainties ($U$, Equation S2) derived using a coverage factor of k=2, corresponding to an approximate 95% confidence interval, and relative expanded uncertainty ($U_{rel}$, Equation S3) reported for each measurement.^2,3^

$\boldsymbol{u}_{\boldsymbol{A}}\boldsymbol{=}\frac{\boldsymbol{SD}}{\sqrt{\boldsymbol{n}}}$	(*Eq S1*)

$\boldsymbol{U=}\boldsymbol{u}_{\boldsymbol{A}}\boldsymbol{\times k}$	(*Eq S2*)

$\boldsymbol{U}_{\boldsymbol{rel}}\left( \boldsymbol{\%} \right)\boldsymbol{=}\frac{\boldsymbol{U}}{\bar{\boldsymbol{x}}}\boldsymbol{\times100}$	(*Eq S3*)

where $SD$ is the standard deviation of replicate measurements, $n$ is the number of replicates, $k$ is the coverage factor, and $\bar{x}$ is the mean of replicate measurements.

Trueness was evaluated by calculating the bias ($b$, Equation S4), representing the systematic deviation of the measured value from the reference value. Since the PS-Eu NPs and PS MPs are not certified reference materials, the information provided by the manufacturer was used as the reference. Bias was calculated from the average apparent recovery ($Rec$, Equation S5).^4,5^

$\boldsymbol{b}\left( \boldsymbol{\%} \right)\boldsymbol{=Rec}\left( \boldsymbol{\%} \right)\boldsymbol{-100}$	*(Eq S4)*

$\boldsymbol{Rec}\left( \boldsymbol{\%} \right)\boldsymbol{=}\frac{\bar{\boldsymbol{x}}}{\boldsymbol{x}_{\boldsymbol{ref}}}$	*(Eq S5)*

where $\bar{x}$ is the mean of replicate measurements and $x_{ref}$ is the manufacturer-reported value for each measurand.

Table S2. ICP-MS instrumental parameters used for the determination of total element concentrations.

| Instrument configuration | | |
| --- | --- | --- |
| Nebulizer | Teflon MiraMist | |
| Spray chamber | Quartz double-spray Scott type | |
| Torch injector inner diameter | 2.5 mm | |
| Sampling and skimmer cones | Nickel | |
| ICP-MS conditions | | |
| RF plasma power | 1550 W | |
| Plasma gas flow rate | 15 L/min | |
| Auxiliary gas flow rate | 0.9 L/min | |
| Carrier gas flow rate | 1.05 L/min | |
| Sampling depth | 8 mm | |
| Sample uptake flow rate | 0.3 mL/min | |
| Cell parameters | | |
| He gas flow | 0 L/min | 4.5 L/min |
| Octopole bias | -8 V | -18 V |
| Octopole RF | 180 V | 200 V |
| Energy discrimination | 5.0 V | 5.0 V |
| Data acquisition | | |
| Isotopes monitored | ^140^Ce, ^151^Eu, ^153^Eu, ^165^Ho, ^175^Lu | ^140^Ce, ^151^Eu, ^153^Eu ^165^Ho, ^175^Lu |
| Data acquisition mode | Spectrum | |
| Replicates | 5 | |
| Isotopes of internal standards | ^72^Ge, ^103^Rh, ^193^Ir, ^209^Bi | |

**Table S3.** Performance of the analytical procedure (i.e., microwave-assisted digestion followed by ICP-MS analysis) for determining the total mass concentration of Eu in PS-Eu NPs and Ce, Eu, Ho, and Lu in PS MPs stock standards. Accuracy was determined as the recovery between the measured and expected element mass concentrations of the digested ionic standards, and repeatability was calculated as a relative standard deviation of three replicates.

| **Sample** | | **Accuracy (%)** | **Repeatability (%)** |
| --- | --- | --- | --- |
| **PS-Eu NPs** | ^151^Eu 50 ng/mL | 97.9 | 4.7 |
|  | ^153^Eu 50 ng/mL | 98.1 | 4.6 |
| **PS MPs** | ^140^Ce 100 ng/mL | 101.6 | 2.1 |
|  | ^151^Eu 100 ng/mL | 104.5 | 2.1 |
|  | ^153^Eu 100 ng/mL | 104.0 | 2.1 |
|  | ^165^Ho 100 ng/mL | 103.1 | 2.4 |
|  | ^175^Lu 100 ng/mL | 102.7 | 2.1 |

**Table S4.** The inter-day reproducibility of the sample uptake flow rate and transport efficiency was evaluated for the analysis of PS-Eu NPs and PS MPs using the spICP-QMS instrument. Transport efficiency was determined using ‘particle size’ and ‘particle frequency’ methods. Measurement uncertainty is expressed as expanded uncertainty and relative expanded uncertainty, calculated with a coverage factor of k = 2. The reported values are derived from replicate measurements performed on nine separate days for PS-Eu NPs and on three separate days for PS MPs.

| **spICP-QMS** | | **Mean value** | **SD** | **Expanded uncertainty (k=2)** | **Relative expanded uncertainty (%,)** |
| --- | --- | --- | --- | --- | --- |
| **PS-Eu NPs** | Sample uptake (mL/min) | 0.336 | 0.007 | **0.005** | **1.48** |
|  | TE based on ‘size method’ | 0.087 | 0.011 | **0.007** | **8.36** |
|  | TE based on ‘frequency method’ | 0.071 | 0.009 | **0.006** | **8.86** |
| **PS MPs** | Sample uptake (mL/min) | 0.033 | 0.002 | **0.002** | **4.78** |
|  | TE based on ‘size method’ | 0.103 | 0.012 | **0.008** | **7.60** |
|  | TE based on ‘frequency method’ | 0.114 | 0.006 | **0.004** | **3.58** |

**Table S5.** The intra-day repeatability and trueness of PS-Eu NPs measurements evaluated for particle size, particle mass, particle mass concentration and particle number concentration, as determined using spICP-QMS and spICP-HRMS. The reported values are based on three (spICP-QMS) or two (spICP-HRMS) replicate measurements.

| ICP-MS | Measurand | **Uncertainty** | | | | **Trueness** | | |
| --- | --- | --- | --- | --- | --- | --- | --- | --- |
|  |  | Mean | SD | Expanded uncertainty (k=2) | **Relative expanded uncertainty (%)** | Reference data | Recovery (%) | **Bias (%)** |
| spICP-QMS | Size (nm) | 196 | 0.77 | 0.89 | **0.5** | 198^a^ | 98.9 | **-1.10** |
|  | Mass (ag) | 4,298 | 50 | 58 | **1.4** | 4,306^b^ | 99.8 | **-0.19** |
|  | Mass concentration (mg/L) | 9,197 | 240 | 277 | **3.0** | 10,000^a^ | 92.0 | **-8.03** |
|  | Number concentration (particles/mL) | 2.282E+12 | 3.638E+10 | 4.200E+10 | **1.8** | 2.322E+12^a^ | 98.3 | **-1.73** |
| spICP-HRMS | Size (nm) | 201 | 0.26 | 0.37 | **0.2** | 198^a^ | 101.7 | **1.7** |
|  | Mass (ag) | 4,585 | 21 | 30 | **0.7** | 4,306^b^ | 106.5 | **6.5** |
|  | Mass concentration (mg/L) | 9,074 | 42 | 59 | **0.7** | 10,000^a^ | 90.7 | **-9.3** |
|  | Number concentration (particles/mL) | 3.862E+12 | 3.588E+10 | 1.487E+10 | **1.3** | 2.322E+12^a^ | 166.3 | **66.3** |

^a^ as provided by a supplier

^b^ calculated by assuming spherical particles with known particle diameter (198 nm) and polystyrene density (1.06 g/cm^3^)

**Table S6.** The inter-day reproducibility and trueness of PS-Eu NPs measurements evaluated for particle size, particle mass, particle mass concentration and particle number concentration, as determined using spICP-QMS. The reported values are based on measurements conducted over nine separate days, comprising a total of 38 replicate measurements.

| spICP-QMS | **Uncertainty** | | | | **Trueness** | | |
| --- | --- | --- | --- | --- | --- | --- | --- |
|  | Mean | SD | Expanded uncertainty (k=2) | **Relative expanded uncertainty (%)** | Reference data | Recovery (%) | **Bias (%)** |
| Size (nm) | 201 | 8 | 3 | **1.34** | 198^a^ | 101.7 | **1.66** |
| Mass (ag) | 4,549 | 569 | 185 | **4.06** | 4,306^b^ | 105.7 | **5.65** |
| Mass concentration (mg/L) | 9,669 | 939 | 305 | **3.15** | 10,000^a^ | 96.7 | **-3.31** |
| Number concentration (particles/mL) | 2.50E+12 | 2.73E+11 | 8.87E+10 | **3.55** | 2.322E+12^a^ | 107.5 | **7.50** |

^a^ as provided by a supplier

^b^ calculated by assuming spherical particles with known particle diameter (198 nm) and polystyrene density (1.06 g/cm^3^)

Table S7. Comparison of the results obtained with spICP-QMS and spICP-HRMS analysis of PS-Eu NPs, and processed by using SPCal and vendors' software. The reported values represent the average ± standard deviation of three (spICP-QMS) or two (spICP-HRMS) replicate measurements.

| Technique | Software | Mean particle diameter (nm) | Mean particle mass (ag) | Particle mass concentration (mg/L) | Particle number concentration (particles/mL) |
| --- | --- | --- | --- | --- | --- |
| spICP-QMS | SPCal | 196 ± 1 | 4,298 ± 50 | 9,197 ± 240 | (2.282 ± 0.036) × 10^12^ |
|  | Vendor | 198 ± 1 | 4,321 ± 35 | 9,412 ± 269 | (2.426 ± 0.047) × 10^12^ |
| spICP-HRMS | SPCal | 201 ± 1 | 4,585 ± 21 | 9,074 ± 42 | (3.862 ± 0.036) × 10^12^ |
|  | Vendor | 203 ± 1 | 4,615 ± 35 | 9,142 ± 22 | (3.891 ± 0.021) × 10^12^ |
| Reference data | | 198^a^ / 184 ± 2^b^ | 4,306^c^ | 10,000^a^ / (9,202 ± 984)^d^ | 2,322 × 10^12^ ^a^ |

^a^ as provided by a supplier

^b^ as determined by NTA

^c^ calculated by assuming spherical particles with known particle diameter (198 nm) and polystyrene density (1.06 g/cm^3^)

^d^ total PS concentration as determined by py-GC-MS

**Table S8.** Transport efficiency determined with the vendor’s software and applied for nanoplastics and microplastics analysis using spICP-QMS or spICP-HRMS. The reported standard deviation for TE (spICP-QMS) represents the measurement repeatability within a single day.

| **Transport efficiency** | **spICP-QMS vendor’s software** | **spICP-HRMS vendor’s software** |
| --- | --- | --- |
| Nanoplastics | Size: 7.90% ± 0.00  Frequency: 6.85% ± 0.05% | Size: 4.97%  Frequency: 2.54% |
| Microplastics | Size: 10.45% ± 0.05%  Frequency: 10.95% ± 0.55% | Size: 8.95%  Frequency: 0.59% |

**Figure S1.** Time scans showing the ^13^C signal of individual PS particles measured with spICP-QMS and spICP-HRMS. The signal-to-background ratio (S/B) was calculated as a ratio between the particle signal intensity and mean background intensity. In spICP-HRMS analysis, the carbon particle signal intensity was 1.7 times higher than in spICP-QMS, while the carbon background was substantially elevated—approximately fourfold higher (36.53 counts vs. 8.88 counts), resulting in a higher S/B ratio for spICP-QMS.

**Table S9.** The intra-day repeatability and trueness of PS MPs measurements evaluated for particle size, particle mass, particle mass concentration and particle number concentration, as determined using spICP-QMS and spICP-HRMS. The reported values are based on four (spICP-QMS) or three (spICP-HRMS) replicate measurements.

| Element | ICP-MS | Measurand | **Uncertainty** | | | | **Trueness** | | |
| --- | --- | --- | --- | --- | --- | --- | --- | --- | --- |
|  |  |  | Mean | SD | Expanded uncertainty (k=2) | **Relative Uncertainty (%)** | Reference data | Recovery (%) | **Bias (%)** |
| **Ce** | **spICP-QMS** | Size (nm) | 109.5 | 0.35 | 0.35 | **0.3** | 109.7^a^ | 99.8 | **-0.2** |
|  |  | Mass (ag) | 4,687 | 40 | 40 | **0.8** | 4,626^a^ | 101.3 | **1.3** |
|  |  | Mass concentration (µg/L) | 0.659 | 0.057 | 0.057 | **8.7** | 1.375^c^ | 47.9 | **-52.1** |
|  |  | Number concentration (particles/mL) | 137,914 | 13,081 | 13,081 | **9.5** | 330,000^a^ | 41.8 | **-58.2** |
|  | **spICP-HRMS** | Size (nm) | 101.6 | 0.46 | 0.54 | **0.5** | 109.7^a^ | 92.6 | **-7.4** |
|  |  | Mass (ag) | 3,760 | 52 | 60 | **1.6** | 4,626^a^ | 81.3 | **-18.7** |
|  |  | Mass concentration (µg/L) | 0.118 | 0.005 | 0.006 | **4.8** | 1.375^c^ | 8.6 | **-91.4** |
|  |  | Number concentration (particles/mL) | 249,847 | 9,311 | 10,751 | **4.3** | 330,000^a^ | 75.7 | **-24.3** |
| **Eu** | **spICP-QMS** | Size (nm) | 121.9 | 0.10 | 0.10 | **0.1** | 101.1^a^ | 120.5 | **20.5** |
|  |  | Mass (ag) | 4,986 | 12 | 12 | **0.2** | 2,833^a^ | 176.0 | **76.0** |
|  |  | Mass concentration (µg/L) | 0.743 | 0.052 | 0.052 | **7.0** | 1.547^c^ | 48.0 | **-52.0** |
|  |  | Number concentration (particles/mL) | 146,076 | 10,443 | 10,443 | **7.1** | 330,000^a^ | 44.3 | **-55.7** |
|  | **spICP-HRMS** | Size (nm) | 122.4 | 0.31 | 0.36 | **0.3** | 101.1^a^ | 121.1 | **21.1** |
|  |  | Mass (ag) | 5,095 | 38 | 44 | **0.9** | 2,833^a^ | 179.8 | **79.8** |
|  |  | Mass concentration (µg/L) | 0.153 | 0.005 | 0.006 | **4.0** | 1.547^c^ | 9.9 | **-90.1** |
|  |  | Number concentration (particles/mL) | 237,724 | 9,340 | 10,785 | **4.5** | 330,000^a^ | 72.0 | **-28.0** |
| **Ho** | **spICP-QMS** | Size (nm) | 70.7 | 0.12 | 0.12 | **0.2** | 76.8^a^ | 92.0 | **-8.0** |
|  |  | Mass (ag) | 1,631 | 9 | 9 | **0.6** | 2,082^a^ | 78.3 | **-21.7** |
|  |  | Mass concentration (µg/L) | 0.235 | 0.011 | 0.011 | **4.5** | 0.549^c^ | 42.8 | **-57.2** |
|  |  | Number concentration (particles/mL) | 141,213 | 6,849 | 6,849 | **4.9** | 330,000^a^ | 42.8 | **-57.2** |
|  | **spICP-HRMS** | Size (nm) | 73.2 | 0.36 | 0.42 | **0.6** | 76.8^a^ | 95.3 | **-4.7** |
|  |  | Mass (ag) | 1,820 | 29 | 33 | **1.8** | 2,082^a^ | 87.4 | **-12.6** |
|  |  | Mass concentration (µg/L) | 0.062 | 0.002 | 0.002 | **3.3** | 0.549^c^ | 11.3 | **-88.7** |
|  |  | Number concentration (particles/mL) | 269,350 | 3,944 | 4,555 | **1.7** | 330,000^a^ | 81.6 | **-18.4** |
| **Lu** | **spICP-QMS** | Size (nm) | 75.1 | 0.18 | 0.18 | **0.2** | 82.1^a^ | 91.5 | **-8.5** |
|  |  | Mass (ag) | 2,190 | 17 | 17 | **0.8** | 2,848^a^ | 76.9 | **-23.1** |
|  |  | Mass concentration (µg/L) | 0.319 | 0.021 | 0.021 | **6.5** | 0.794^c^ | 40.1 | **-59.9** |
|  |  | Number concentration (particles/mL) | 142,625 | 9,691 | 9,691 | **6.8** | 330,000^a^ | 43.2 | **-56.8** |
|  | **spICP-HRMS** | Size (nm) | 75.1 | 0.23 | 0.26 | **0.3** | 82.1^a^ | 91.5 | **-8.5** |
|  |  | Mass (ag) | 2,208 | 21 | 24 | **1.1** | 2,848^a^ | 77.5 | **-22.5** |
|  |  | Mass concentration (µg/L) | 0.071 | 0.005 | 0.005 | **7.6** | 0.794^c^ | 8.9 | **-91.1** |
|  |  | Number concentration (particles/mL) | 254,064 | 14,965 | 17,280 | **6.8** | 330,000^a^ | 77.0 | **-23.0** |
| **C** | **spICP-QMS** | Size (nm) | 2,890 | 22 | 22 | **0.8** | 2,500^a^ | 115.6 | **15.6** |
|  |  | Mass (ag) | 1.43E+07 | 3.09E+05 | 3.09E+05 | **2.2** | 8.67E+06^b^ | 164.6 | **64.6** |
|  |  | Mass concentration (µg/L) | 2,185 | 180 | 180 | **8.2** | 2,860^d^ | 76.4 | **-23.6** |
|  |  | Number concentration (particles/mL) | 149,849 | 9,255 | 9,255 | **6.2** | 330,000^a^ | 45.4 | **-54.6** |
|  | **spICP-HRMS** | Size (nm) | 2,940 | 42 | 48 | **1.6** | 2,500^a^ | 117.6 | **17.6** |
|  |  | Mass (ag) | 1.45E+07 | 5.89E+05 | 6.81E+05 | **4.7** | 8.67E+06^b^ | 167.6 | **67.6** |
|  |  | Mass concentration (µg/L) | 468 | 49 | 57 | **12.2** | 2,860^d^ | 16.4 | **-83.6** |
|  |  | Number concentration (particles/mL) | 255,118 | 18,275 | 21,102 | **8.3** | 330,000^a^ | 77.3 | **-22.7** |

^a^ as provided by a supplier/literature data

^b^ calculated by assuming spherical particles with known particle diameter (2500 nm) and polystyrene density (1.06 g/cm^3^)

^c^ total element concentration as determined by conventional ICP-MS analysis of the acid-digested stock solution

^d^ calculated by multiplying the particles number concentration as provided by a supplier with the mean particle mass

**Table S10.** The inter-day reproducibility and trueness of PS MPs measurements evaluated for particle size, particle mass, particle mass concentration and particle number concentration, determined using spICP-QMS. The reported values are based on measurements conducted over three separate days, comprising a total of 16 (Ce, Eu, Ho, Lu) and 28 (C) replicate measurements.

| Element | spICP-QMS | **Uncertainty** | | | | **Trueness** | | |
| --- | --- | --- | --- | --- | --- | --- | --- | --- |
|  |  | Mean | SD | Expanded uncertainty (k=2) | **Relative expanded uncertainty (%)** | Reference data | Recovery (%) | **Bias (%)** |
| **Ce** | Size (nm) | 109.5 | 0.42 | 0.21 | **0.19** | 109.7^a^ | 99.8 | **-0.17** |
|  | Mass (ag) | 4,656 | 54 | 27 | **0.58** | 4,626^a^ | 100.6 | **0.64** |
|  | Mass concentration (µg/L) | 0.894 | 0.149 | 0.075 | **8.35** | 1.375^c^ | 65.0 | **-34.97** |
|  | Number concentration (particles/mL) | 157,711 | 17,239 | 8,620 | **5.47** | 330,000^a^ | 47.8 | **-52.21** |
| **Eu** | Size (nm) | 119.6 | 1.08 | 0.54 | **0.45** | 101.1^a^ | 118.3 | **18.27** |
|  | Mass (ag) | 4,700 | 127 | 64 | **1.35** | 2,833^a^ | 165.9 | **65.89** |
|  | Mass concentration (µg/L) | 0.983 | 0.159 | 0.080 | **8.09** | 1.547^c^ | 63.5 | **-36.49** |
|  | Number concentration (particles/mL) | 171,416 | 20,397 | 10,198 | **5.95** | 330,000^a^ | 51.9 | **-48.06** |
| **Ho** | Size (nm) | 68.3 | 1.15 | 0.58 | **0.84** | 76.8^a^ | 88.9 | **-11.13** |
|  | Mass (ag) | 1,466 | 75 | 38 | **2.57** | 2,082^a^ | 70.4 | **-29.58** |
|  | Mass concentration (µg/L) | 0.305 | 0.046 | 0.023 | **7.51** | 0.549^c^ | 55.5 | **-44.46** |
|  | Number concentration (particles/mL) | 171,278 | 21,813 | 10,907 | **6.37** | 330,000^a^ | 51.9 | **-48.10** |
| **Lu** | Size (nm) | 71.4 | 1.93 | 0.97 | **1.36** | 82.1^a^ | 86.9 | **-13.06** |
|  | Mass (ag) | 1,878 | 157 | 79 | **4.18** | 2,848^a^ | 65.9 | **-34.06** |
|  | Mass concentration (µg/L) | 0.400 | 0.056 | 0.028 | **6.99** | 0.794^c^ | 50.3 | **-49.68** |
|  | Number concentration (particles/mL) | 176,105 | 24,872 | 12,436 | **7.06** | 330,000^a^ | 53.4 | **-46.63** |
| **C** | Size (nm) | 2,853 | 24 | 9 | **0.32** | 2,500^a^ | 114.1 | **14.13** |
|  | Mass (ag) | 1.29E+07 | 3.30E+05 | 1.25E+05 | **0.97** | 8.67E+06^b^ | 148.7 | **48.73** |
|  | Mass concentration (µg/L) | 2,609 | 615 | 233 | **8.92** | 2,860^d^ | 91.2 | **-8.79** |
|  | Number concentration (particles/mL) | 163,929 | 24,163 | 9,133 | **5.57** | 330,000^a^ | 49.7 | **-50.32** |

^a^ as provided by a supplier/literature data

^b^ calculated by assuming spherical particles with known particle diameter (2500 nm) and polystyrene density (1.06 g/cm^3^)

^c^ total element concentration as determined by conventional ICP-MS analysis of the acid-digested stock solution

^d^ calculated by multiplying the particles number concentration as provided by a supplier with the mean particle mass

Table S11. Comparison of results obtained with spICP-QMS and spICP-HRMS analysis of lanthanide-doped PS MPs processed by using SPCal and vendors' software. The reported values represent the average ± standard deviation of four (spICP-QMS) or three (spICP-HRMS) replicate measurements.

| **Monitored isotope** | **Technique (Software)** | **Mean particle diameter (nm)** | **Mean particle mass (ag)** | **Particle mass concentration (μg/L)** | **Particle number concentration (particles/mL)** |
| --- | --- | --- | --- | --- | --- |
| Ce | spICP-QMS (SPCal) | 109.5 ± 0.4 | 4,687 ± 40 | 0.659 ± 0.057 | 137,914 ± 13,081 |
|  | spICP-QMS (Vendor) | 109.1 ± 0.3 | 4,601 ± 35 | 0.663 ± 0.058 | 137,233 ± 12,979 |
|  | spICP-HRMS (SPCal) | 101.6 ± 0.5 | 3,760 ± 52 | 0.118 ± 0.005 | 249,847 ± 9,311 |
|  | spICP-HRMS (Vendor) | 112.2 ± 0.6 | 5,003 ± 79 | 0.116 ± 0.005 | 257,792 ± 7,658 |
|  | Reference data | 109.7^a^ | 4,626^a^ | 1.375 ± 0.064^c^ | 330,000 ^a^ |
| Eu | spICP-QMS (SPCal) | 121.9 ± 0.1 | 4,986 ± 12 | 0.743 ± 0.052 | 146,076 ± 10,443 |
|  | spICP-QMS (Vendor) | 121.3 ± 0.1 | 4,905 ± 13 | 0.749 ± 0.054 | 145,277 ± 10,870 |
|  | spICP-HRMS (SPCal) | 122.4 ± 0.3 | 5,095 ± 38 | 0.153 ± 0.005 | 237,724 ± 9,340 |
|  | spICP-HRMS (Vendor) | 130.5 ± 0.2 | 6,105 ± 29 | 0.139 ± 0.005 | 253,533 ± 10,244 |
|  | Reference data | 101.1^a^ | 2,833^a^ | 1.547 ± 0.045^c^ | 330,000 ^a^ |
| Ho | spICP-QMS (SPCal) | 70.7 ± 0.1 | 1,631 ± 9 | 0.235 ± 0.011 | 141,213 ± 6,849 |
|  | spICP-QMS (Vendor) | 70.2 ± 0.1 | 1,595 ± 8 | 0.238 ± 0.010 | 141,808 ± 6,639 |
|  | spICP-HRMS (SPCal) | 73.2 ± 0.4 | 1,820 ± 29 | 0.062 ± 0.002 | 269,350 ± 3,944 |
|  | spICP-HRMS (Vendor) | 80.9 ± 0.2 | 2,437 ± 18 | 0.061 ± 0.002 | 277,083 ± 5,261 |
|  | Reference data | 76.8^a^ | 2,082^a^ | 0.549 ± 0.022^c^ | 330,000 ^a^ |
| Lu | spICP-QMS (SPCal) | 75.1 ± 0.2 | 2,190 ± 17 | 0.319 ± 0.021 | 142,625 ± 9,691 |
|  | spICP-QMS (Vendor) | 74.7 ± 0.1 | 2,148 ± 12 | 0.321 ± 0.021 | 142,129 ± 9,591 |
|  | spICP-HRMS (SPCal) | 75.1 ± 0.2 | 2,208 ± 21 | 0.071 ± 0.005 | 254,064 ± 14,965 |
|  | spICP-HRMS (Vendor) | 80.9 ± 0.1 | 2,729 ± 11 | 0.066 ± 0.003 | 268,500 ± 14,544 |
|  | Reference data | 82.1^a^ | 2,848^a^ | 0.794 ± 0.007^c^ | 330,000 ^a^ |
| C | spICP-QMS (SPCal) | 2,890 ± 22 | (1.44 ± 0.03) × 10^7^ | 2,185 ± 180 | 149,849 ± 9,255 |
|  | spICP-QMS (Vendor) | 2,879 ± 24 | (1.32 ± 0.03) × 10^7^ | 2,253 ± 181 | 152,098 ± 8,732 |
|  | spICP-HRMS (SPCal) | 2,940 ± 42 | (1.45 ± 0.06) × 10^7^ | 468 ± 49 | 255,118 ± 18,275 |
|  | spICP-HRMS (Vendor) | 3,142 ± 56 | (1.72 ± 0.09) × 10^7^ | 435 ± 48 | 279,833 ± 17,434 |
|  | Reference data | 2,500^a^ | 8.67 × 10^6^ ^b^ | 2,860^d^/ (1,837 ± 82) ^e^ | 330,000 ^a^ |

^a^ as provided by a supplier/literature data

^b^ calculated by assuming spherical particles with known particle diameter (2500 nm) and polystyrene density (1.06 g/cm^3^)

^c^ total element concentration as determined by conventional ICP-MS analysis of the acid-digested stock solution

^d^ calculated by multiplying the particles number concentration as provided by a supplier with the mean particle mass

^e^ total PS concentration as determined by py-GC-MS

**References**

1 V. Barwick, Ed., *Eurachem Guide: Terminology in Analytical Measurement – Introduction to VIM 3*, Second., 2023.
2 JCGM, *Evaluation of measurement data-Guide to the expression of uncertainty in measurement Évaluation des données de mesure-Guide pour l’expression de l’incertitude de mesure*, 2008.
3 U. S. Ellison Secretary, R. Bettencourt da Silva, E. P. Poland Fodor, R. Kaarls, E. B. Germany Magnusson, I. P. Robouch, E. St Gallen, S. A. van der Veen, M. W. Walsh Eurachem IRE Wegscheider, P. Yolci Omeroglu and E. Representatives, *EURACHEM/CITAC Guide Quantifying Uncertainty in Analytical Measurement Composition of the Working Group* EURACHEM members A Williams Chairman, A. Brzyski, R. Kaus, E. Amico di Meane, M. Rösslein, A. Fajgelj, IAEA Vienna*, 2009.
4 International Organization for Standardization, *ISO 21748:2017 Guidance for the use of repeatability, reproducibility and trueness estimates in measurement uncertainty evaluation*, 2017.
5 International Organization for Standardization, *ISO 5725-1:2023 Accuracy (trueness and precision) of measurement methods and results Part 1: General principles and definitions*, 2023.
